# Efficacy and safety of atezolizumab plus bevacizumab treatment for advanced hepatocellular carcinoma in the real world: a single-arm meta-analysis

**DOI:** 10.1186/s12885-023-11112-w

**Published:** 2023-07-06

**Authors:** Xiaoqiang Gao, Rui Zhao, Huaxing Ma, Shi Zuo

**Affiliations:** 1 Department of Hepatobiliary Surgery, Affiliated Hospital of Guizhou Medical University, 28 Guiyi Street, Yunyan District, Guizhou 550000 Guiyang, China; 2 Department of Emergency Surgery, Affiliated Hospital of Guizhou Medical University, Guiyang, Guizhou China

**Keywords:** Hepatocellular carcinoma, Atezolizumab, Bevacizumab, Meta-analysis, Single-arm

## Abstract

**Background:**

Atezolizumab plus bevacizumab was approved in 2020 as a first-line treatment for advanced hepatocellular carcinoma (HCC). The purpose of this study was to assess the curative effect and tolerability of the combination treatment in advanced HCC.

**Methods:**

Web of Science, PubMed and Embase were retrieved for qualified literatures on the treatment of advanced HCC with atezolizumab plus bevacizumab until September 1, 2022. The outcomes included pooled overall response (OR), complete response (CR), partial response (PR), median overall survival (mOS), median progression-free survival (mPFS), and adverse events (AEs).

**Results:**

Twenty-three studies, comprising 3168 patients, were enrolled. The pooled OR, CR, and PR rates of the long-term (more than six weeks) therapy response based on Response Evaluation Criteria in Solid Tumors (RECIST) were 26%, 2%, and 23%, respectively. The pooled OR, CR, and PR rates of the short-term (six weeks) therapeutic response evaluated with RECIST were 13%, 0%, and 15%, respectively. The pooled mOS and mPFS were 14.7 months and 6.66 months, respectively. During the treatment, 83% and 30% of patients experienced any grade AEs and grade 3 and above AEs, respectively.

**Conclusions:**

Atezolizumab in combination with bevacizumab showed good efficacy and tolerability in the treatment of advanced HCC. Compared with short-term, non-first-line, and low-dose therapy, atezolizumab plus bevacizumab in long-term, first-line, and standard-dose treatment for advanced HCC showed a better tumor response rate.

**Supplementary Information:**

The online version contains supplementary material available at 10.1186/s12885-023-11112-w.

## Background

Hepatocellular carcinoma (HCC) is the most frequent type of liver cancer, representing approximately 90% of cases [1]. The major known risk factors for HCC are viral, metabolic, toxic, and immune system-related diseases [2]. Although the successful implementation of the hepatitis B virus (HBV) vaccine program has reduced the incidence of HCC among middle-aged people (30–59 years), the incidence and mortality of HCC have continued to rise due to the non-alcoholic fatty liver disease pandemic. Studies demonstrated that the global HCC mortality rate would increase another 41% by 2040 [3, 4]. Despite advances in HCC diagnosis, a majority of individuals with HCC are identified at advanced stages since the illness is typically asymptomatic in the early stages [5]. The treatment choices for HCC differ depending on the liver function, tumor load, physical condition, and comorbidities of a patient. For early-stage HCC, the primary therapeutic approaches are surgical resection and radiofrequency ablation [6, 7]. Transarterial chemoembolization serves as a standard of therapy for intermediate-stage HCC, and systemic anticancer strategies are the main treatment for advanced HCC [8, 9]. Over the last decade, sorafenib, lenvatinib, and regorafenib, the molecular target agents, have been utilized as the main drugs in the treatment of advanced HCC. However, these agents offer limited survival advantages [10].

Immune checkpoint inhibitors (ICIs), which can activate the anti-tumor activity of immune cells, have revolutionized cancer therapy, and single-agent ICIs show a response rate of 15–20% for HCC treatment [11, 12]. Because vascular endothelial growth factor (VEGF) is a key element in the formation of an immunosuppressive tumor microenvironment (TME) in addition to promoting tumor angiogenesis, VEGF blockade and ICI combination therapy may be more effective for HCC characterized by high vascularization [13]. In May 2020, atezolizumab (an ICI) in combination with bevacizumab (a VEGF monoclonal antibody) was authorized as a new first-line treatment for advanced HCC based on the phase III IMbrave150 trial [14, 15]. The external validity of randomized controlled trial (RCT) results is generally low, and the outcomes of RCTs on atezolizumab combined with bevacizumab in advanced HCC are inconsistent; further research is needed to determine the real utility of the combination treatment in clinical practice [15–17]. In the past two years, many relevant studies have emerged, and their cumulative analysis may contribute to the preliminary clinical validation of the RCT results [15, 17]. The meta-analysis was performed to estimate the efficacy and safety of the combination treatment of atezolizumab and bevacizumab for advanced HCC in the real world.

## Methods

### Data sources and search strategy

This study followed the PRISMA declaration and was registered at PROSPERO (CRD42022377004) [18]. Two independent researchers (XG and RZ) searched Web of Science, PubMed, and EMBASE for eligible papers published before September 1, 2022. The search strategy used was (“atezolizumab” OR “anti-PDL1” OR “MPDL3280A” OR “Tecentriq” OR “RG7446”) AND (“Bevacizumab” OR “Mvasi” OR “Avastin” OR “Bevacizumab awwb”) AND (“Liver cancer” OR “Hepatocellular Carcinoma” OR “Hepatoma” OR “HCC”). There were no restrictions on the search based on geography, race, age, or payment type. In addition, references of potentially eligible articles were investigated for useful studies.

### Inclusion and exclusion criteria

The criteria for inclusion were as follows: (1) participants: all patients with a definite diagnosis of HCC; (2) interventions: patients were managed with atezolizumab plus bevacizumab; (3) outcomes: at least one clinical tumor outcome, such as overall response (OR), complete response (CR), partial response (PR), median overall survival (mOS), median progression-free survival (mPFS), and adverse events (AEs), was documented in the literature; (4) studies: prospective clinical and retrospective studies published in English, including RCTs, cohort studies, and single-arm studies.

The exclusion criteria were as follows: (1) pathological studies, animal experiments, case reports, reviews, letters, conference abstracts, comments, and editorials; (2) literature in other languages or containing incomplete data; (3) original literature unavailable.

Two investigators (XG and RZ) independently assessed the eligibility of the articles strictly on the basis of the inclusion and exclusion criteria. Any inconsistencies were ironed out with the support of a third investigator (HM).

### Data extraction and quality assessment

Two reviewers (XG and RZ) performed data extraction from included articles independently, and the third reviewer (HM) would join the discussion when ambiguity emerged. The extracted data were as follows: first author, year of publication, research type, nation, sample size, age, intervention method, and reported endpoints. The clinical and safety outcomes were mOS, mPFS, OR, CR, PR, and the occurrence of any AEs and ≥ grade 3 AEs. The quality of the included retrospective single-arm studies was assessed using the JBI Critical Appraisal Checklist for Case Series [19]. The Newcastle-Ottawa Scale was utilized to evaluate the included RCT studies and the retrospective studies with a comparison group [20].

### Statistical analysis

STATA MP 16.0 (Stata Corp., TX, United States) was used for all data analysis, and a double-arcsine transformation was applied when the data did not fit a normal distribution. The 95% CI represents the effect magnitude of all combined results. The I^2^ statistic was employed for the heterogeneity analysis of studies. As for pooling the effect size, the fixed-effects model was applied when I^2^ < 50%, and the random-effects model was used when I^2^ ≥ 50% because of the significant heterogeneity. By removing each study from the pooled results one by one, a sensitivity analysis was conducted. Furthermore, the publication bias in the included studies was assessed with Begg’s and Egger’s tests.

## Results

### Characteristics of the included studies

**Fig. 1:**
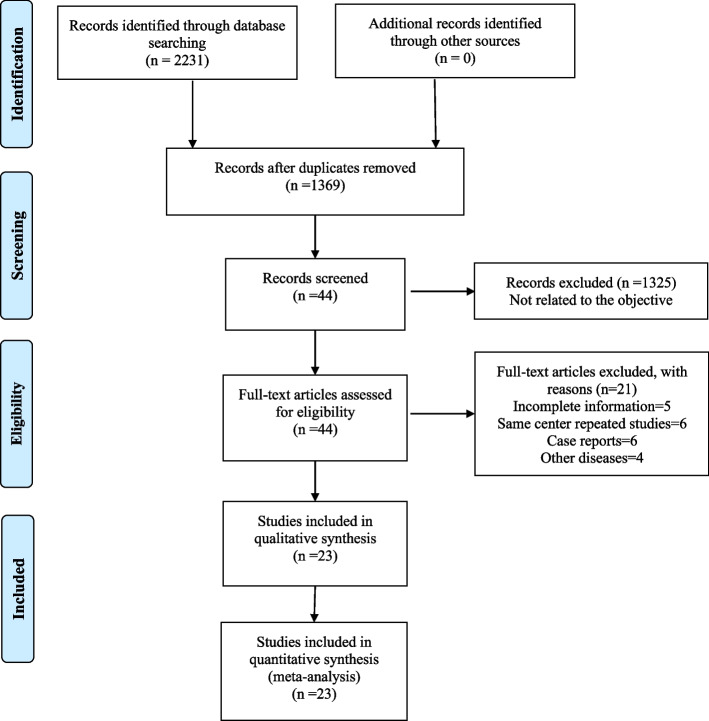
Literature screening process

In total, 2231 relevant publications were located by carefully examining the three electronic databases. After a series of screening and de-duplication steps, 23 studies involving 3168 patients were enrolled in our study [17, 21–42]. Our detailed study screening process is shown in Fig. 1. The general features and quality evaluation results of the 23 studies are displayed in Tables 1 and 2, respectively.

**Table 1:** Baseline clinical characteristics of the included studies

Study	Country	Design	Period	Sample size (male/female)	Age, median, y	Intervention	End points
Castro 2022 [21]	Germany	Retrospective	11/2019-11/2021	147(125/22)	68.7 (30–96)	A 1200 mg + B 15 mg/kg	OR, PR, CR, OS, PFS, AEs
Teng 2022 [22]	Taiwan	Retrospective	09/2020-01/2022	89(75/14)	61.3 (56.4–67.8)	A 1200 mg + B 5-7.5 mg/kg	OR, PR, CR, OS, PFS, AEs
Komatsu 2022 [23]	Japan	Retrospective	10/2020-01/2021	34(25/9)	73 (45–82)	A 1200 mg + B 15 mg/kg	OR, PR, CR, AEs
Kuzuya 2022 [24]	Japan	Retrospective	10/2020-07/2021	50(44/6)	73 (38–85)	A 1200 mg + B 15 mg/kg	OR, PR, CR, PFS, AEs
Cheon 2022 [25]	South Korea	Retrospective	05/2020-02/2021	121(101/20)	61 (36–83)	A 1200 mg + B 15 mg/kg	OR, PR, CR, PFS, AEs
Himmelsbach 2022 [26]	Germany	Retrospective	12/2018-08/2021	66(54/12)	65 (30–88)	A 1200 mg + B 15 mg/kg	OR, PR, CR, OS, PFS, AEs
Maesaka 2022 [27]	Japan	Retrospective	10/2020-05/2021	88(71/17)	75 (47–91)	A 1200 mg + B 15 mg/kg	OR, PR, CR, PFS, AEs
Iwamoto 2021 [28]	Japan	Retrospective	11/2020-03/2021	51(45/6)	71 (37–85)	A 1200 mg + B 15 mg/kg	OR, PR, CR, PFS, AEs
Tomonari 2022 [29]	Japan	Retrospective	09/2020-09/2021	71(58/13)	71 (66–79)	A 1200 mg + B 15 mg/kg	OR, PR, CR, PFS, AEs
Sho 2022 [30]	Japan	Retrospective	10/2020-02/2022	115(95/20)	72 (31–89)	A 1200 mg + B 15 mg/kg	OR, PR, CR, PFS
Chon 2022 [31]	Korea	Retrospective	05/2020-04/2021	121(100/21)	63 (57–71)	A 1200 mg + B 15 mg/kg	OR, PR, CR, PFS, AEs
D’Alessio 2022 [32]	UK	Retrospective	01/2019-01/2022	202(173/29)	69 (23–90)	A 1200 mg + B 15 mg/kg	OR, PR, CR, OS, PFS, AEs
Eso 2021 [33]	Japan	Retrospective	10/2020-08/2021	40(35/5)	70.5 (53–82)	A 1200 mg + B 15 mg/kg	OR, PR, CR, PFS, AEs
Chen 2022 [34]	Taiwan	Retrospective	01/2018-05/2021	41(38/3)	65 (23–83)	A 1200 mg + B 15 mg/kg	OR, OS
Fulgenzi 2022 [35]	UK	Retrospective	01/2019-01/2022	296(245/51)	66 (59–73)	A 1200 mg + B 15 mg/kg	OR, PR, CR, OS, PFS, AEs
Tada 2022 [36]	Japan	Retrospective	09/2020-10/2021	317(258/59)	74 (68–80)	A 1200 mg + B 15 mg/kg	OR, PR, CR, OS, PFS, AEs
Chuma 2022 [37]	Japan	Retrospective	10/2020-06/2021	94(73/21)	73 (37–87)	A 1200 mg + B 15 mg/kg	OR, PR, CR, AEs
Wang 2022 [38]	Taiwan	Retrospective	01/2020-10/2021	48(38/10)	62 (31–80)	A 1200 mg + B 5-7.5 mg/kg	OR, PR, CR, PFS, AEs
Tanaka 2022 [39]	Japan	Retrospective	09/2020-03/2022	457(368/89)	74 (68–79)	A 1200 mg + B 15 mg/kg	AEs
Kim 2022 [40]	South Korea	Retrospective	08/2019-07/2021	86(70/16)	62 (56–71)	A 1200 mg + B 15 mg/kg	OR, PR, CR, PFS, AEs
Hiraoka 2022 [41]	Japan	Retrospective	01/2020-01/2022	194(148/46)	74 (68–79)	A 1200 mg + B 15 mg/kg	OR, PR, CR, PFS, AEs
Cheng 2022 [42]	USA	Clinical trial (NCT03434379)	03/2018-08/2020	336(227/109)	64 (56–71)	A 1200 mg + B 15 mg/kg	OR, PR, CR, OS, PFS, AEs
Lee 2020 [17]	South Korea	Clinical trial (NCT02715531)	07/2016-06/2019	104(84/20)	62 (23–82)	A 1200 mg + B 15 mg/kg	OR, PR, CR, OS, PFS, AEs

*A *Atezolizumab, *B *Bevacizumab, *OR *Overall response, *PR *Partial response, *CR *Complete response, *OS *Overall survival, *PFS *Progression-free survival, *AEs  A*dverse events

**Table 2:** Quality assessment of included studies

**A. The JBI Critical Appraisal Checklist for Case Series for included retrospective single-arm studies**											
**Study**	**Q1**	**Q2**	**Q3**	**Q4**	**Q5**	**Q6**	**Q7**	**Q8**	**Q9**	**Q10**	**Overall appraisal**
Castro 2022 [21]	Yes	Yes	Yes	No	Yes	Yes	Yes	Yes	Yes	Yes	Include
Teng 2022 [22]	Yes	Yes	Yes	Yes	Yes	Yes	Yes	Yes	No	Yes	Include
Komatsu 2022 [23]	Yes	Yes	Yes	Yes	Yes	Yes	Yes	Yes	Yes	Yes	Include
Kuzuya 2022 [24]	Yes	Yes	Yes	Yes	Yes	Yes	Yes	Yes	Yes	Yes	Include
Cheon 2022 [25]	Yes	Yes	Yes	Yes	Yes	Yes	Yes	Yes	Yes	Yes	Include
Himmelsbach 2022 [26]	Yes	Yes	Yes	Yes	Yes	Yes	Yes	Yes	Yes	Yes	Include
Maesaka 2022 [27]	Yes	Yes	Yes	Yes	Yes	Yes	Yes	Yes	Yes	Yes	Include
Iwamoto 2021 [28]	Yes	Yes	Yes	Yes	Yes	Yes	Yes	Yes	Yes	Yes	Include
Tomonari 2022 [29]	Yes	Yes	Yes	Yes	Yes	Yes	Yes	Yes	Yes	Yes	Include
Sho 2022 [30]	Yes	Yes	Yes	Yes	Yes	Yes	Yes	Yes	Yes	Yes	Include
Chon 2022 [31]	Yes	Yes	Yes	Yes	Yes	Yes	Yes	Yes	Yes	Yes	Include
D’Alessio 2022 [32]	Yes	Yes	Yes	No	Yes	Yes	Yes	Yes	Yes	Yes	Include
Eso 2021 [33]	Yes	Yes	Yes	Yes	Yes	Yes	Yes	Yes	Yes	Yes	Include
Chen 2022 [34]	Yes	Yes	Yes	Yes	Yes	Yes	Yes	Yes	Yes	Yes	Include
Fulgenzi 2022 [35]	Yes	Yes	Yes	Yes	Yes	Yes	Yes	Yes	No	Yes	Include
Tada 2022 [36]	Yes	Yes	Yes	Unclear	Yes	Yes	Yes	Yes	Yes	Yes	Include
Chuma 2022 [37]	Yes	Yes	Yes	Unclear	Yes	Yes	Yes	Yes	Yes	Yes	Include
Wang 2022 [38]	Yes	Yes	Yes	Yes	Yes	Yes	Yes	Yes	Yes	Yes	Include
Tanaka 2022 [39]	Yes	Yes	Yes	Yes	Yes	Yes	Yes	Yes	Yes	Yes	Include
**B. The Newcastle–Ottawa Scale was used to assess the other suitable studies**											
**Study**		**Selection**			**Comparability**			**Outcome**			**Quality score**
Kim 2022 [40]		4			2			2			8
Hiraoka 2022 [41]		4			2			2			8
Cheng 2022 [42]		4			2			3			9
Lee 2020 [17]		4			2			3			9

#### Numbers Q1-Q10 in heading signified

Q1, were there clear criteria for inclusion in the case series? Q2, was the condition measured in a standard, reliable way for all participants included in the case series? Q3, were valid methods used for identification of the condition for all participants included in the case series? Q4, did the case series have consecutive inclusion of participants? Q5, did the case series have complete inclusion of participants? Q6, was there clear reporting of the demographics of the participants in the study? Q7, was there clear reporting of clinical information of the participants? Q8, were the outcomes or follow up results of cases clearly reported? Q9, was there clear reporting of the presenting site(s)/clinic(s) demographic information? Q10, was statistical analysis appropriate?.

### Tumor response

**Fig. 2:**
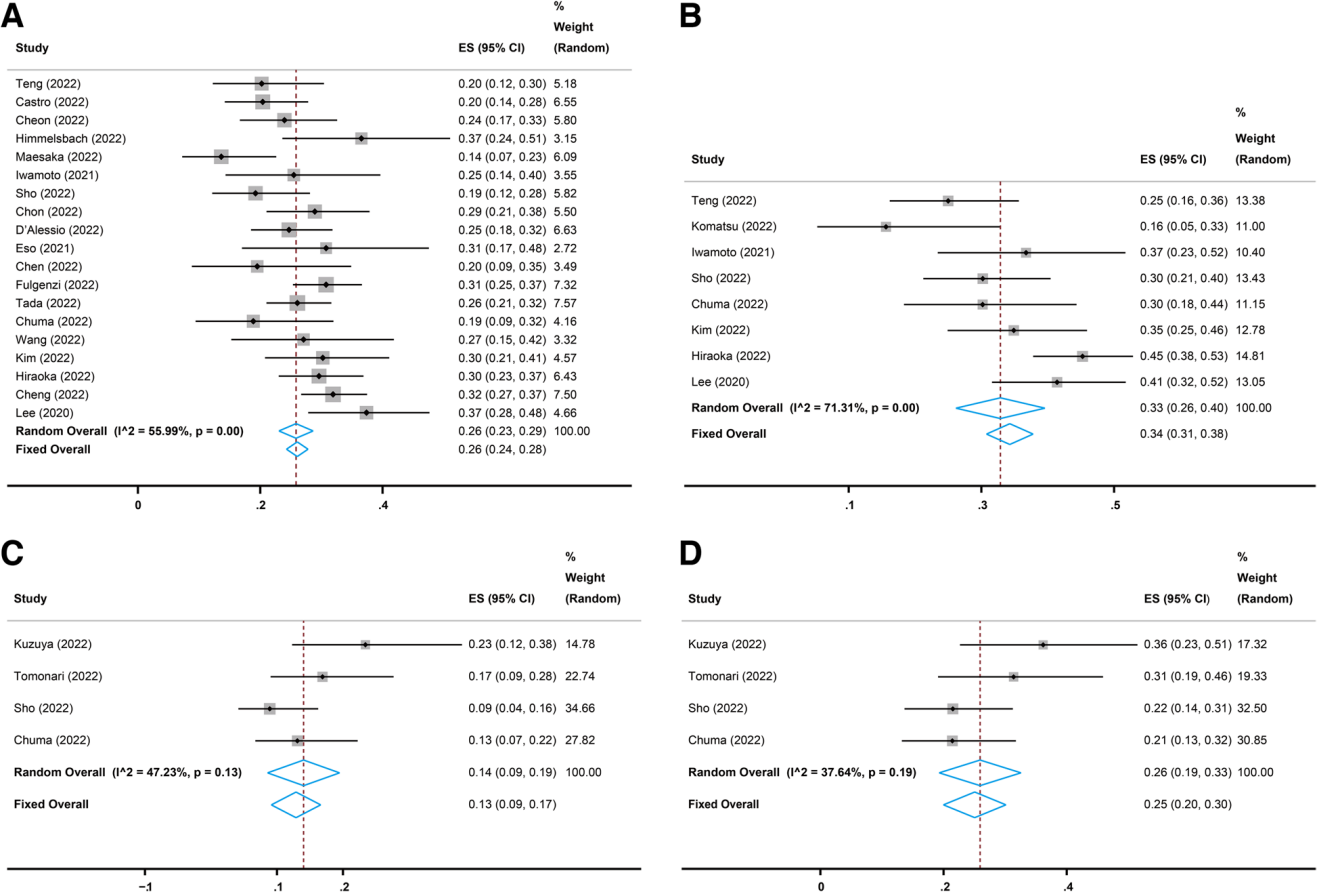
Pooled overall response (OR) rates of short-term and long-term treatment with atezolizumab plus bevacizumab. **A** Pooled OR rate of long-term treatment based on Response Evaluation Criteria in Solid Tumors (RECIST). **B** Pooled OR rate of long-term treatment based on modified RECIST (mRECIST). **C** Pooled OR rate of short-term treatment based on RECIST. **D** Pooled OR rate of short-term treatment based on mRECIST

**Fig. 3:**
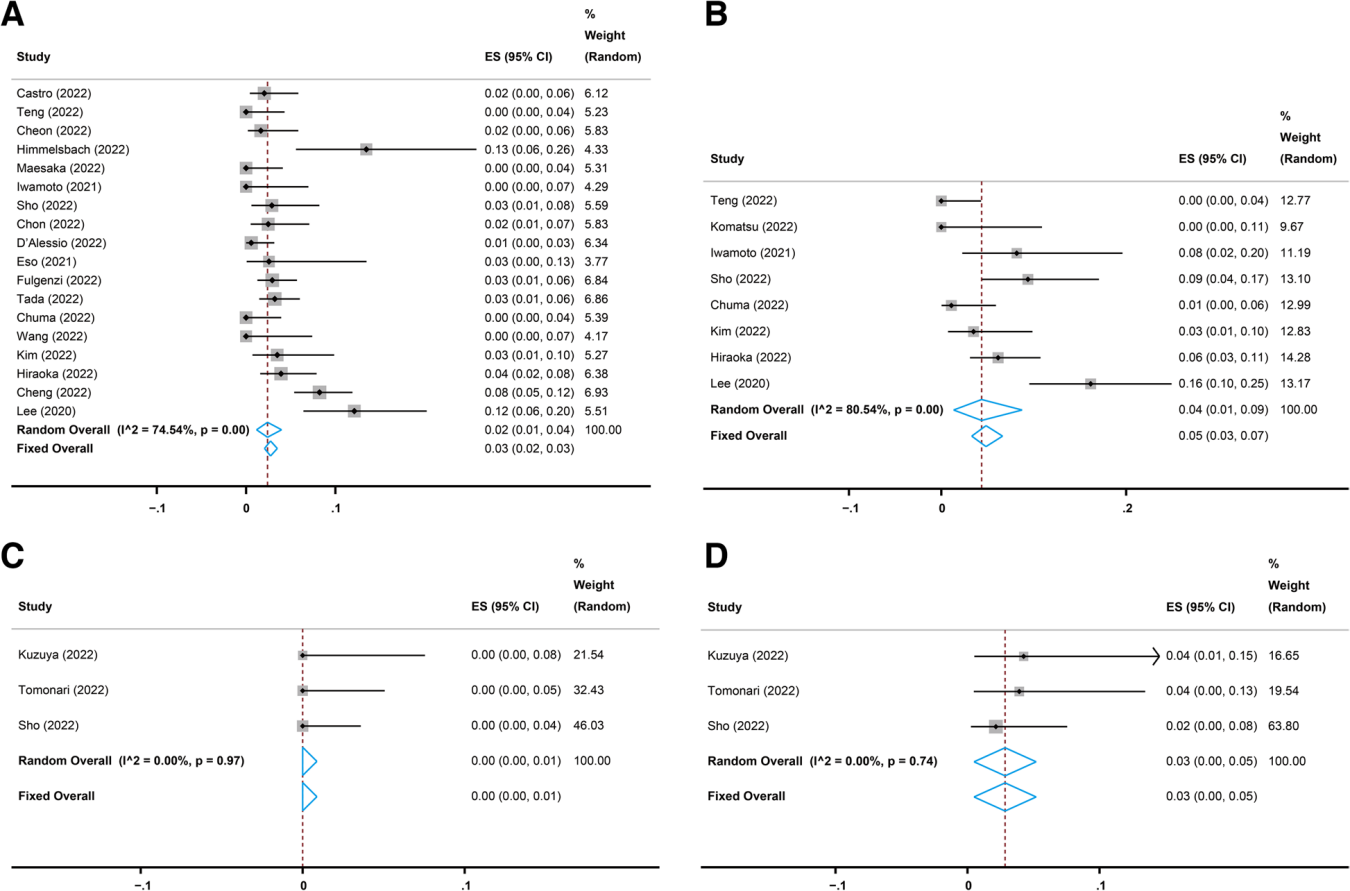
Pooled complete response (CR) rates of short-term and long-term treatment with atezolizumab plus bevacizumab. **A** Pooled CR rate of long-term treatment based on Response Evaluation Criteria in Solid Tumors (RECIST). **B** Pooled CR rate of long-term treatment based on modified RECIST (mRECIST). (**C**) Pooled CR rate of short-term treatment based on RECIST. **D** Pooled CR rate of short-term treatment based on mRECIST

**Fig. 4:**
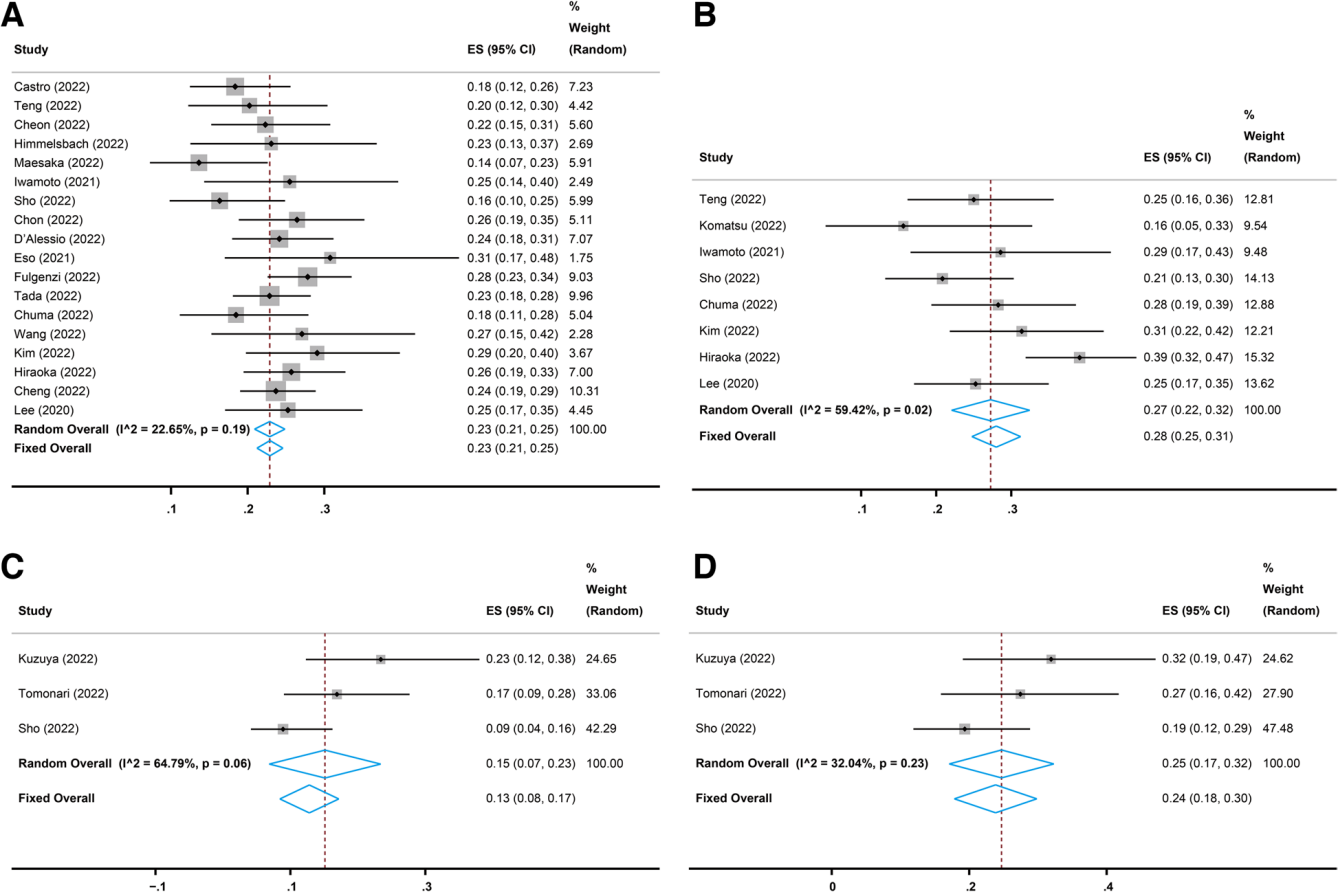
Pooled partial response (PR) rates of short-term and long-term treatment with atezolizumab plus bevacizumab. **A** Pooled PR rate of long-term treatment based on Response Evaluation Criteria in Solid Tumors (RECIST). **B** Pooled PR rate of long-term treatment based on modified RECIST (mRECIST). **C** Pooled PR rate of short-term treatment based on RECIST. **D** Pooled PR rate of short-term treatment based on mRECIST

Nineteen studies [17, 21, 22, 25–28, 30–38, 40–42] reported the long-term (more than six weeks) performance of the combination treatment of atezolizumab and bevacizumab in advanced HCC. All 19 studies reported the long-term OR following atezolizumab plus bevacizumab therapy evaluated with Response Evaluation Criteria in Solid Tumors (RECIST), and the pooled OR rate was 26% (95% CI, 23–29%, I^2^ = 55.99%, *p* = 0.00, Fig. 2A). Eighteen [17, 21, 22, 25–28, 30–33, 35–38, 40–42] of the 19 studies reported the long-term CR and PR evaluated with RECIST, and the pooled CR and PR rates were 2% (95% CI, 1–4%, I^2^ = 74.54%, *p* = 0.00, Fig. 3A) and 23% (95% CI, 21–25%, I^2^ = 22.65%, *p* = 0.19, Fig. 4A), respectively. In addition, eight [17, 22, 23, 28, 30, 37, 40, 41] of the 19 studies reported the long-term OR, CR, and PR evaluated with modified RECIST (mRECIST), and the pooled OR rate was 33% (95% CI, 26–40%, I^2^ = 71.31%, *p* = 0.00, Fig. 2B), while the pooled CR and PR rates were 4% (95% CI, 1–9%, I^2^ = 80.54%, *p* = 0.00, Fig. 3B) and 27% (95% CI, 22–32%, I^2^ = 59.42%, *p* = 0.02, Fig. 4B), respectively.

Four studies [24, 29, 30, 37] reported the short-term (six weeks) performance of atezolizumab combined with bevacizumab in advanced HCC. All four studies reported the short-term OR evaluated with RECIST, and the pooled OR rate was 13% (95% CI, 9–17%, I^2^ = 47.23%, *p* = 0.13, Fig. 2C). Three [24, 29, 30] of the four studies reported the short-term CR and PR evaluated with RECIST, and the pooled CR and PR rates were 0% (95% CI, 0–1%, I^2^ = 0.00%, *p* = 0.97, Fig. 3C) and 15% (95% CI, 7–23%, I^2^ = 64.79%, *p* = 0.06, Fig. 4C), respectively. Moreover, all four studies reported the short-term OR evaluated with mRECIST, and the pooled OR rate was 25% (95% CI, 20–30%, I^2^ = 37.64%, *p* = 0.19, Fig. 2D), while three [24, 29, 30] of the four studies reported the short-term CR and PR evaluated with mRECIST, and the pooled CR and PR rates were 3% (95% CI, 0–5%, I^2^ = 0.00%, *p* = 0.74, Fig. 3D) and 24% (95% CI, 18–30%, I^2^ = 32.04%, *p* = 0.23, Fig. 4D), respectively.

Four studies [22, 33, 37, 38] reported the performance of atezolizumab in combination with bevacizumab on the basis of the treatment lines. In the first-line treatment of the combination, the pooled OR rates were 29% (95% CI, 22–37%, I^2^ = 0.00%, *p* = 0.43, Supplemental Fig. 1A) and 35% (95% CI, 27–44%, I^2^ = 0.00%, *p* = 0.98, Supplemental Fig. 1B), the pooled CR rates were 0% (95% CI, 0–2%, I^2^ = 0.00%, *p* = 0.93, Supplemental Fig. 2A) and 2% (95% CI, 0–17%, I^2^ = 71.26%, *p* = 0.03, Supplemental Fig. 2B), and the pooled PR rates were 26% (95% CI, 20–32%, I^2^ = 22.23%, *p* = 0.28, Supplemental Fig. 3A) and 29% (95% CI, 19–40%, I^2^ = 0.00%, *p* = 0.56, Supplemental Fig. 3B) when evaluated with RECIST and mRECIST, respectively. In the second-line and above treatments, the pooled OR rates were 13% (95% CI, 8–19%, I^2^ = 0.00%, *p* = 0.42, Supplemental Fig. 1C) and 19% (95% CI, 9–28%, I^2^ = 55.38%, *p* = 0.08, Supplemental Fig. 1D), the pooled CR rates were 0% (95% CI, 0–4%, I^2^ = 0.00%, *p* = 0.44, Supplemental Fig. 2C) and 0% (95% CI, 0–4%, I^2^ = 0.00%, *p* = 0.51, Supplemental Fig. 2D), and the pooled PR rates were 16% (95% CI, 8–23%, I^2^ = 0.00%, *p* = 0.59, Supplemental Fig. 3C) and 17% (95% CI, 6–29%, I^2^ = 60.17%, *p* = 0.08, Supplemental Fig. 3D) when evaluated with RECIST and mRECIST, respectively.

The IMbrave150 trial’s inclusion criteria were as follows: no prior systemic therapy for advanced HCC; a Child-Pugh score (CPS) of A; and the score of the Eastern Cooperative Oncology Group (ECOG) was 0 or 1 [15]. Six studies [17, 21, 25, 35, 37, 42] reported the OR of atezolizumab combined with bevacizumab treatment in the IMbrave-IN group who fulfilled the inclusion criteria, and the pooled OR rate evaluated with RECIST was 30% (95% CI, 28–33%, I^2^ = 5.13%, *p* = 0.38, Supplemental Fig. 4A). Two studies [21, 37] reported the OR in the IMbrave-OUT group who met at least one of the exclusion criteria of the IMbrave150 study (patients receiving the combination treatment of atezolizumab and bevacizumab as non-first-line therapy, a CPS of B, or ECOG ≥ 2) [21, 37], and the pooled OR rate assessed with RECIST was 13% (95% CI, 6–19%, I^2^ = 0.00%, Supplemental Fig. 4B). Seventeen studies [17, 21, 25–28, 30–37, 40–42] reported the OR of the standard-dose therapy (1200 mg of atezolizumab plus 15 mg/kg of bevacizumab) [15], and the pooled OR rate assessed with RECIST was 26% (95% CI, 23–29%, I^2^ = 59.03%, *p* = 0.00, Supplemental Fig. 4C). Two studies [22, 38] reported the OR of the low-dose therapy (1200 mg of atezolizumab plus 5–7.5 mg/kg of bevacizumab), and the pooled OR rate evaluated with RECIST was 22% (95% CI, 15–30%, I^2^ = 0.00%, Supplemental Fig. 4D).

### Survival

**Fig. 5:**
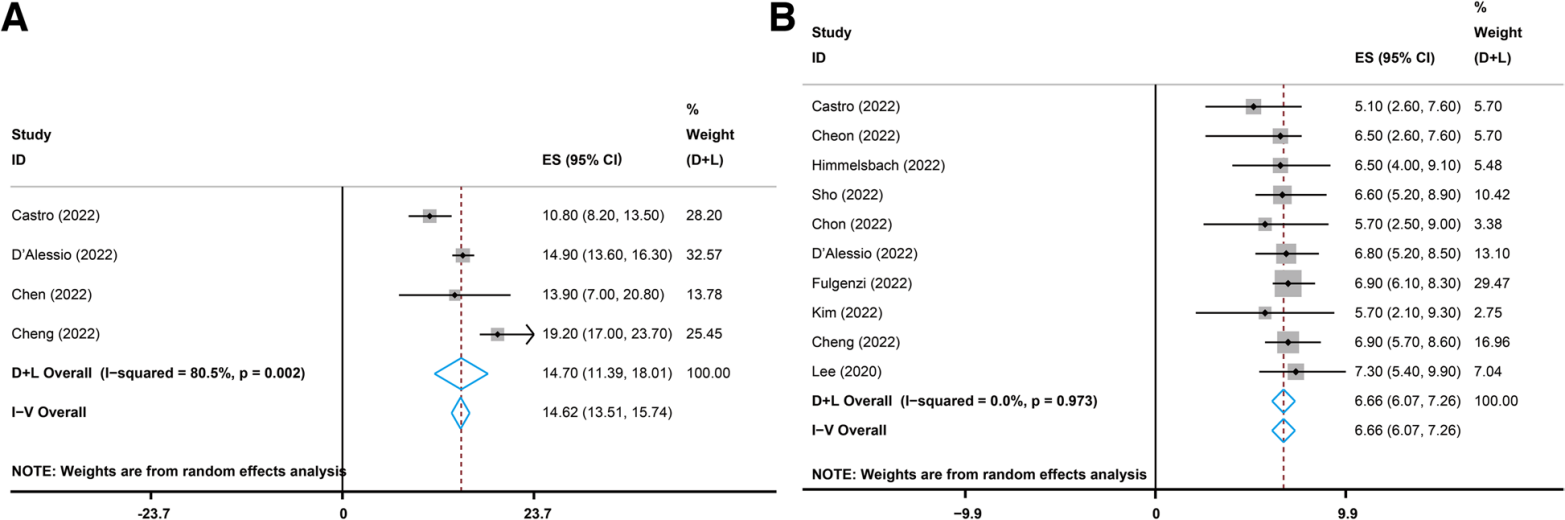
Pooled results of the median overall survival (mOS) and median progression-free survival (mPFS) with atezolizumab plus bevacizumab. **A** Pooled results of the mOS. **B** Pooled results of the mPFS

Four studies [21, 32, 34, 42] reported the complete mOS data for the combination treatment, and the pooled mOS was 14.70 months (95% CI, 11.39–18.01, I^2^ = 80.5%, *p* = 0.002, Fig. 5A). Ten studies [17, 21, 25, 26, 30–32, 35, 40, 42] reported the complete mPFS data, and the pooled mPFS was 6.66 months (95% CI, 6.07–7.26, I2 = 0.0%, *p* = 0.973, Fig. 5B).

### Toxicity

**Fig. 6:**
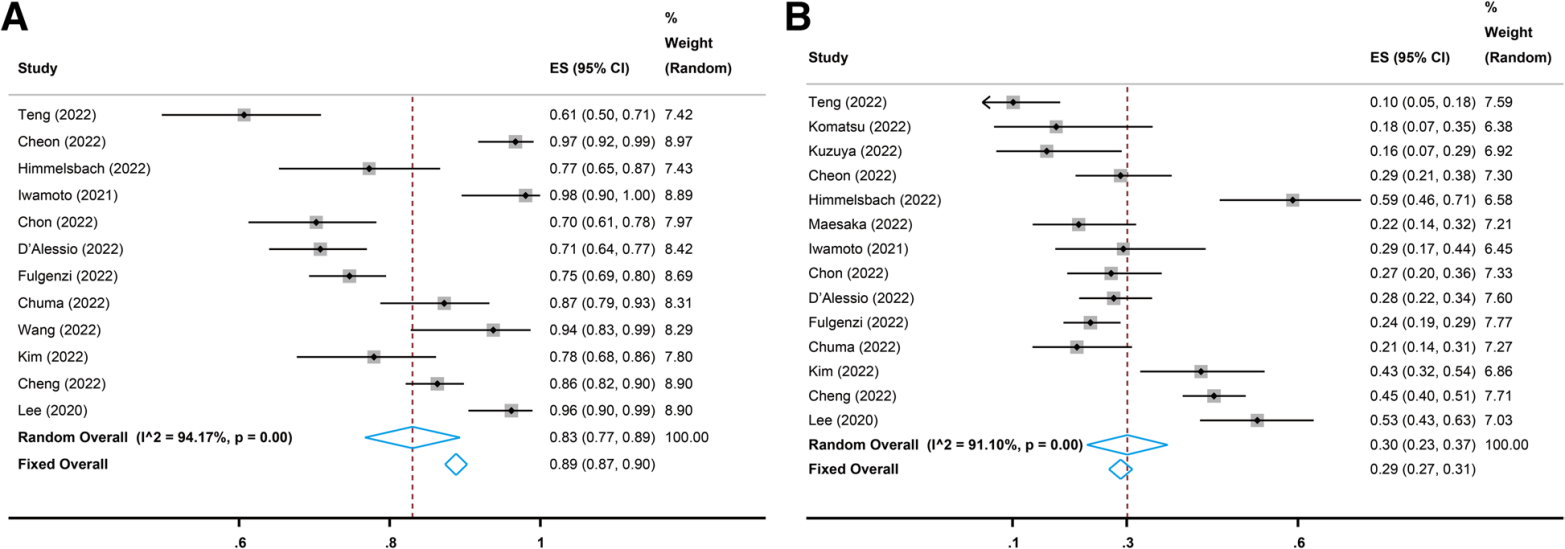
Pooled incidence of all-grade and grade 3 and above adverse events (AEs). **A** Pooled incidence of the all-grade AEs. **B** Pooled incidence of the grade 3 and above AEs

Twelve studies [17, 22, 25, 26, 28, 31, 32, 35, 37, 38, 40, 42] reported the incidence of all-grade AEs, and the pooled incidence was 83% (95% CI, 77–89%, I^2^ = 94.17%, *p* = 0.00, Fig. 6A). The most common all-grade AEs included aspartate transaminase (AST) increase, alanine aminotransferase (ALT) elevation, proteinuria, hypertension, fatigue, thrombocytopenia, appetite loss, pyrexia, peripheral edema, pruritus, nausea, rash, and blood bilirubin increase (Table 3). Fourteen studies [17, 22–28, 31, 32, 35, 37, 40, 42] reported the incidence of grade ≥ 3 AEs, and the pooled incidence was 30% (95% CI, 23–37%, I^2^ = 91.10%, *p* = 0.00, Fig. 6B). The most common grade 3 and above AEs included AST increase, hypertension, proteinuria, ALT elevation, gastrointestinal hemorrhage, thrombocytopenia, blood bilirubin increase, pyrexia, fatigue, pulmonary embolism, pneumonia, and colitis (Table 3).

**Table 3:** Pooled results of common adverse events

Adverse Event	All Grade		≥ Grade 3	
	ES, % (95% CI)	I^2^, %	ES, % (95% CI)	I^2^, %
AST increase	31 (22–40)	92.10	5 (3–7)	55.13
ALT elevation	24 (16–32)	89.08	3 (2–4)	0.00
Proteinuria	24 (18–30)	94.07	4 (3–6)	72.72
Hypertension	24 (19–29)	90.94	5 (3–8)	82.19
Fatigue	23 (20–27)	79.42	1 (0–1)	31.31
Thrombocytopenia	20 (11–29)	96.90	2 (1–4)	59.06
Appetite loss	19 (16–23)	76.14	0 (0–1)	35.49
Pyrexia	17 (10–23)	89.81	1 (0–2)	0.00
Peripheral edema	17 (0–34)	93.97	0 (0–1)	18.37
Pruritus	13 (9–16)	51.63	0 (0–1)	35.36
Nausea	10 (6–14)	75.86	0 (0–1)	0.00
Rash	10 (9–12)	35.05	0 (0–1)	0.00
Blood bilirubin increase	10 (4–16)	92.15	2 (1–3)	13.35
Colitis	8 (5–10)	84.15	1 (0–1)	40.44
Thyroid dysfunction	5 (3–7)	75.73	0 (0–0)	0.00
Gastrointestinal hemorrhage	4 (2–5)	73.19	3 (1–5)	64.59
Pulmonary embolism	1 (0–2)	0.00	1 (0–2)	0.00
Pneumonia	1 (1–2)	0.00	1 (0–1)	0.00
Hand-foot syndrome	1 (1–2)	45.23	0 (0–0)	0.00

*ES *Effect size, *CI *Confidence interval, *AST *Aspartate transaminase, *ALT *Alanine aminotransferase

### Sensitivity analysis

The sensitivity analysis was carried out by deleting each study one by one to determine its impact on the pooled results. The results showed that none of the pooled results were significantly affected by any single study, which indicated that the results of this study are relatively reliable (Supplemental Fig. 5).

### Publication bias

Egger’s and Begg’s tests were used to detect the publication bias in the meta-analysis. The evaluation results showed no significant publication bias for the pooled short-term OR evaluated with RECIST (Egger’s test and Begg’s test: *p* = 0.823 and 0.944, respectively) and the pooled incidence of grade 3 and above AEs (Egger’s test and Begg’s test: *p* = 0.616 and *p* = 0.584, respectively), while demonstrating the existence of publication bias for the pooled incidence of all-grade AEs (Egger’s test and Begg’s test: *p* = 0.009 and 0.115, respectively) and the pooled mPFS (Egger’s test and Begg’s test: *p* = 0.017 and 0.025, respectively) in the included studies.

## Discussion

The first-line treatment of sorafenib has been the standard therapy for advanced HCC since 2007 [42]. Systemic therapy for HCC is constantly evolving; lenvatinib was authorized as a first-line treatment in 2018 [42]; in 2020, in light of the significant improvement in the prognosis of HCC patients in the IMbrave150 trial, atezolizumab in combination with bevacizumab was approved as the first-line treatment for advanced HCC [15]. This meta-analysis explored the curative effect and tolerability of atezolizumab combined with bevacizumab in the treatment of advanced HCC in clinical practice based on the latest published studies.

Among all 23 included studies, the number of female patients was significantly lower than that of male patients, which may be related to the higher incidence of HCC in males [1]. In this meta-analysis, the performance of atezolizumab plus bevacizumab therapy in HCC for 6 weeks reported in some studies was defined as short-term performance [24, 29, 30, 37], while the therapeutic effect for more than 6 weeks reported in some research was defined as long-term performance [17, 21, 22, 25–28, 30–38, 40–42]. Our study revealed that regardless of prior therapies, disease status, and drug dosage, the short-term and long-term OR rates of the combination treatment of atezolizumab and bevacizumab for advanced HCC according to RECIST were 13% and 26%, respectively, with corresponding CR rates of 0% and 2% and corresponding PR rates of 15% and 23%. The long-term combination therapy was demonstrated to deliver great performance in the entire group of patients with advanced HCC; however, the short-term efficacy was unsatisfactory. In this research, we defined the drug dosage for the combination therapy used in the IMbrave150 trial (1200 mg of atezolizumab plus 15 mg/kg of bevacizumab) as the standard dose, while the drug dosage recommended in Taiwan’s Patient Support Program (PSP) (1200 mg of atezolizumab plus 5–7.5 mg/kg of bevacizumab) was defined as the low dose [15, 22]. The OR rate was 26% for standard-dose therapy and 22% for low-dose therapy based on RECIST; the corresponding CR rates were 3% and 0%, and the corresponding PR rates were 23% and 22%, respectively, which indicated that the drug dosage had a certain influence on the efficacy of the combination treatment. Therefore, physicians can actively refer to the standard dosage suggested in the IMbrave150 trial in clinical practice [15]. Our analysis results also found that the OR (29%) and PR (26%) rates of the first-line treatment were significantly higher than the OR (13%) and PR (16%) rates of the second-line and above treatments evaluated with RECIST, respectively, and also higher than the corresponding outcomes of the conventional first-line sorafenib [42]. Although the tumor response rate of atezolizumab plus bevacizumab as non-first-line therapy is much lower than that of the first-line treatment of the combination, it is higher than that of the second-line drugs currently used for advanced HCC (regorafenib, cabozantinib, and ramucirumab) [12]. Hence, this combination therapy may serve as a non-first-line treatment for advanced HCC patients who are resistant to other conventional systemic treatments in the future, but more clinical research is required. Furthermore, based on RECIST, the pooled OR rate of the IMbrave-IN group was 30%, which is not only consistent with the result of the IMbrave150 trial but also higher than that of the long-term treatment group (26%), standard dosage treatment group (26%), and first-line treatment group (29%), which might be attributable to the strict inclusion criteria in the IMbrave150 trial [15, 42]. However, the OR rate was relatively low in the IMbrave-OUT group (16%). Our results tested the inclusion criteria, dosage, and tumor response for the combination therapy of atezolizumab and bevacizumab in the IMbrave150 trial, further confirming its authority as a clinical guideline for the treatment of advanced HCC. Apart from the high tumor response rate, the pooled mOS (14.7 months) and mPFS (6.66 months) of HCC patients receiving atezolizumab plus bevacizumab were significantly prolonged compared to sorafenib or lenvatinib, although not as long as those reported in the IMbrave150 trial [40, 42].

Atezolizumab can reactivate T cell cytotoxicity by blocking PD-L1 from binding to PD-1 or A7-1 receptors [43], and the single-agent OR rate was 17% in patients with advanced HCC in a phase Ib study [44]. Bevacizumab can suppress angiogenesis and tumor growth, and the single-agent OR rate was 14% in a phase II study [45]. The monotherapy effects of these two agents are unsatisfactory. Immune evasion and angiogenesis are usually interdependent and occur concurrently in the TME [46]. Preclinical studies provide a solid theoretical basis for the combination immunotherapy of atezolizumab and bevacizumab [47], and clinical studies ultimately verified the efficacy of this combination therapy [17, 21–42]. A recent study showed that bevacizumab could not only suppress angiogenesis but also inhibit VEGF-mediated regulatory T cell (Treg) proliferation and myeloid cell inflammation and work synergistically with atezolizumab to increase the proportion of CD8 + T cells and dendritic cells in TME, thereby activating anti-tumor immunity and hampering tumor growth [48]. Besides, some studies also showed that the pretreatment platelet-to-lymphocyte (PLR) and neutrophil-to-lymphocyte (NLR) values were always lower in the patients who had better efficacy with the atezolizumab combined with bevacizumab treatment, which might be useful predictors for the combination therapy [31, 33, 38].

As for AEs of atezolizumab in combination with bevacizumab therapy, the pooled incidence of all-grade AEs was 83%, and the common all-grade AEs with an incidence of more than 10% were as follows: AST increase (31%), ALT elevation (24%), proteinuria (24%), hypertension (24%), fatigue (23%), thrombocytopenia (20%), appetite loss (19%), pyrexia (17%), peripheral edema (17%), pruritus (13%), nausea (10%), rash (10%), and a blood bilirubin increase (10%). The incidence of grade ≥ 3 AEs was 30%, and the common grade 3 and above AEs were as follows: AST increase (5%), hypertension (5%), proteinuria (4%), ALT elevation (3%), gastrointestinal hemorrhage (3%), thrombocytopenia (2%), and blood bilirubin increase (2%). Overall, the inclusion criteria varied among the studies, which may be the main reason why the pooled incidences of AEs were not consistent with those in the IMbarve150 trial; however, the combination therapy was still well tolerated compared with sorafenib or lenvatinib [41, 42], and the toxicities maintained consistency with those of each agent: the most common AEs for atezolizumab were increased AST, ALT, and bilirubin concentrations; proteinuria and hypertension were the most common AEs for bevacizumab; and no new additional toxicities were noticed [17]. As a result, before treatment, appropriate patients should be carefully selected. During treatment, biological indicators of patients should be closely monitored, drug dosage and the course of treatment should be adjusted in a timely manner, and various treatment-related AEs should be actively and effectively treated. Only in this manner can the influence of AEs be minimized [49].

There were also some shortcomings in our meta-analysis. Firstly, selection bias might exist since only two RCTs were enrolled. Secondly, we couldn’t make comparisons with other mainstream first-line agents due to the limitations of the included studies. Thirdly, there was significant heterogeneity across studies. Fourthly, the study had publication bias. Finally, some analyses could not be carried out because of the lack of data.

## Conclusions

In this study, we found that regardless of prior therapies, disease status, and drug dosage, the combination of atezolizumab and bevacizumab performed well in the treatment of the whole group of patients with advanced HCC. Furthermore, the long-term, first-line, and standard-dose treatment of the combination showed a better tumor response rate compared with the short-term, non-first-line, and low-dose treatment group. The combination therapy may be used as a non-first-line therapy for advanced HCC resistant to other systemic treatments in the future because its second-line and above treatments showed a high tumor response rate in our study. Our analysis also verified the authority of the IMbrave150 trial as a clinical guideline for the treatment of advanced HCC. In addition, the combination therapy was well tolerated, the toxicities were consistent with those of each drug, and no new extra toxicities were seen.

## Supplementary Information

**Additional file 1:** **Supplementary Fig. 1.** Pooled OR rates of first-line and second- or later-line treatment with atezolizumab plus bevacizumab. (**A**) Pooled OR rate of first-line treatment based on RECIST. (B) Pooled OR rate of first-line treatment based on mRECIST. (C) Pooled OR rate of second- or later-line treatment based on RECIST. (D) Pooled OR rate of second- or later-line based on mRECIST. OR, overall response; RECIST, Response Evaluation Criteria in Solid Tumors; mRECIST, modified RECIST. **Supplementary Fig. 2.** Pooled CR rates of first-line and second- or later-line treatment with atezolizumab plus bevacizumab. (A) Pooled CR rate of first-line treatment based on RECIST. (B) Pooled CR rate of first-line treatment based on mRECIST. (C) Pooled CR rate of second- or later-line treatment based on RECIST. (D) Pooled CR rate of second- or later-line based on mRECIST. CR, complete response; RECIST, Response Evaluation Criteria in Solid Tumors; mRECIST, modified RECIST. **Supplementary Fig. 3.** Pooled PR rates of first-line and second- or later-line treatment with atezolizumab plus bevacizumab. (A) Pooled PR rate of first-line treatment based on RECIST. (B) Pooled PR rate of first-line treatment based on mRECIST. (C) Pooled PR rate of second- or later-line treatment based on RECIST. (D) Pooled PR rate of second- or later-line based on mRECIST. PR, partial response; RECIST, Response Evaluation Criteria in Solid Tumors; mRECIST, modified RECIST. **Supplementary Fig. 4.** Pooled OR rates based on different inclusion criteria and different doses of atezolizumab plus bevacizumab. (A) Pooled OR rate of the IMbrave-IN group based on RECIST. (B) Pooled OR rate of IMbrave-OUT group based on RECIST. (C) Pooled OR rate of standard dose (1200 mg of atezolizumab plus 15 mg/kg of bevacizumab) therapy based on RECIST. (D) Pooled OR rate of low dose (1200 mg of atezolizumab plus 5–7.5 mg/kg of bevacizumab) therapy based on RECIST. OR, overall response; RECIST, Response Evaluation Criteria in Solid Tumors. **Supplementary Fig. 5.** The results of sensitivity analysis. (A) OR rate of non-early treatment based on RECIST. (B) OR rate of non-early treatment based on mRECIST. (C) OR rate of early treatment based on RECIST. (D) OR rate of early treatment based on mRECIST. (E) CR rate of early treatment based on mRECIST. (F) CR rate of non-early treatment based on RECIST. (G) CR rate of non-early treatment based on mRECIST. (H) PR rate of early treatment based on RECIST. (I) PR rate of early treatment based on mRECIST. (J) PR rate of non-early treatment based on RECIST. (K) PR rate of non-early treatment based on mRECIST. (L) Incidence of all-grade AEs. (M) Incidence of grade 3 and above AEs. (N) median overall survival. (O) median progression-free survival. OR, overall response; CR, complete response; PR, partial response; RECIST, Response Evaluation Criteria in Solid Tumors; mRECIST, modified RECIST. AEs, adverse events.

## Data Availability

The corresponding author will respond to reasonable requests for the datasets used in the current work.

## Abbreviations

HCC: Hepatocellular carcinoma
HBV: Hepatitis B virus
ICIs: Immune checkpoint inhibitors
VEGF: Vascular endothelial growth factor
TME: Tumor microenvironment
RCT: Randomized controlled trial
OR: Overall response
CR: Complete response
PR: Partial response
mOS: Median overall survival
mPFS: Median progression-free survival
AEs: Adverse events
RECIST: Response Evaluation Criteria in Solid Tumors
mRECIST: Modified RECIST
CPS: Child-Pugh score
ECOG: Eastern Cooperative Oncology Group
AST: Aspartate transaminase
ALT: Alanine aminotransferase
Treg: Regulatory T cell
Teffs: Effector T cells
AFP: Alpha-fetoprotein
GPC3: Glypican-3
PLR: Platelet-to-lymphocyte
NLR: Neutrophil-to-lymphocyte
