# Supplementary material for: Efficacy and safety of atezolizumab plus bevacizumab treatment for advanced hepatocellular carcinoma in the real world: a single-arm meta-analysis
Source: BMC Cancer. 2023 Jul 6;23:635. doi: 10.1186/s12885-023-11112-w (PMC10327339; doi:10.1186/s12885-023-11112-w)
Supplement: Supplementary file 1 — Additional file 1: Supplementary Fig. 1. Pooled OR rates of first-line and second- or later-line treatment with atezolizumab plus bevacizumab. (A) Pooled OR rate of first-line treatment based on RECIST. (B) Pooled OR rate of first-line treatment based on mRECIST. (C) Pooled OR rate of second- or later-line treatment based on RECIST. (D) Pooled OR rate of second- or later-line based on mRECIST. OR, overall response; RECIST, Response Evaluation Criteria in Solid Tumors; mRECIST, modified RECIST. Supplementary Fig. 2. Pooled CR rates of first-line and second- or later-line treatment with atezolizumab plus bevacizumab. (A) Pooled CR rate of first-line treatment based on RECIST. (B) Pooled CR rate of first-line treatment based on mRECIST. (C) Pooled CR rate of second- or later-line treatment based on RECIST. (D) Pooled CR rate of second- or later-line based on mRECIST. CR, complete response; RECIST, Response Evaluation Criteria in Solid Tumors; mRECIST, modified RECIST. Supplementary Fig. 3. Pooled PR rates of first-line and second- or later-line treatment with atezolizumab plus bevacizumab. (A) Pooled PR rate of first-line treatment based on RECIST. (B) Pooled PR rate of first-line treatment based on mRECIST. (C) Pooled PR rate of second- or later-line treatment based on RECIST. (D) Pooled PR rate of second- or later-line based on mRECIST. PR, partial response; RECIST, Response Evaluation Criteria in Solid Tumors; mRECIST, modified RECIST. Supplementary Fig. 4. Pooled OR rates based on different inclusion criteria and different doses of atezolizumab plus bevacizumab. (A) Pooled OR rate of the IMbrave-IN group based on RECIST. (B) Pooled OR rate of IMbrave-OUT group based on RECIST. (C) Pooled OR rate of standard dose (1200 mg of atezolizumab plus 15 mg/kg of bevacizumab) therapy based on RECIST. (D) Pooled OR rate of low dose (1200 mg of atezolizumab plus 5–7.5 mg/kg of bevacizumab) therapy based on RECIST. OR, overall response; RECIST, Response Evaluation Criteria in S [file 12885_2023_11112_MOESM1_ESM.docx]

Supplementary Material

**Efficacy and safety of atezolizumab plus bevacizumab treatment for advanced hepatocellular carcinoma in the real world: a single-arm meta-analysis**

Xiaoqiang Gao, Rui Zhao, Huaxing Ma, Shi Zuo^*^

*** Correspondence:** Shi Zuo: drzuoshi@gmc.edu.cn

## Supplementary Figures


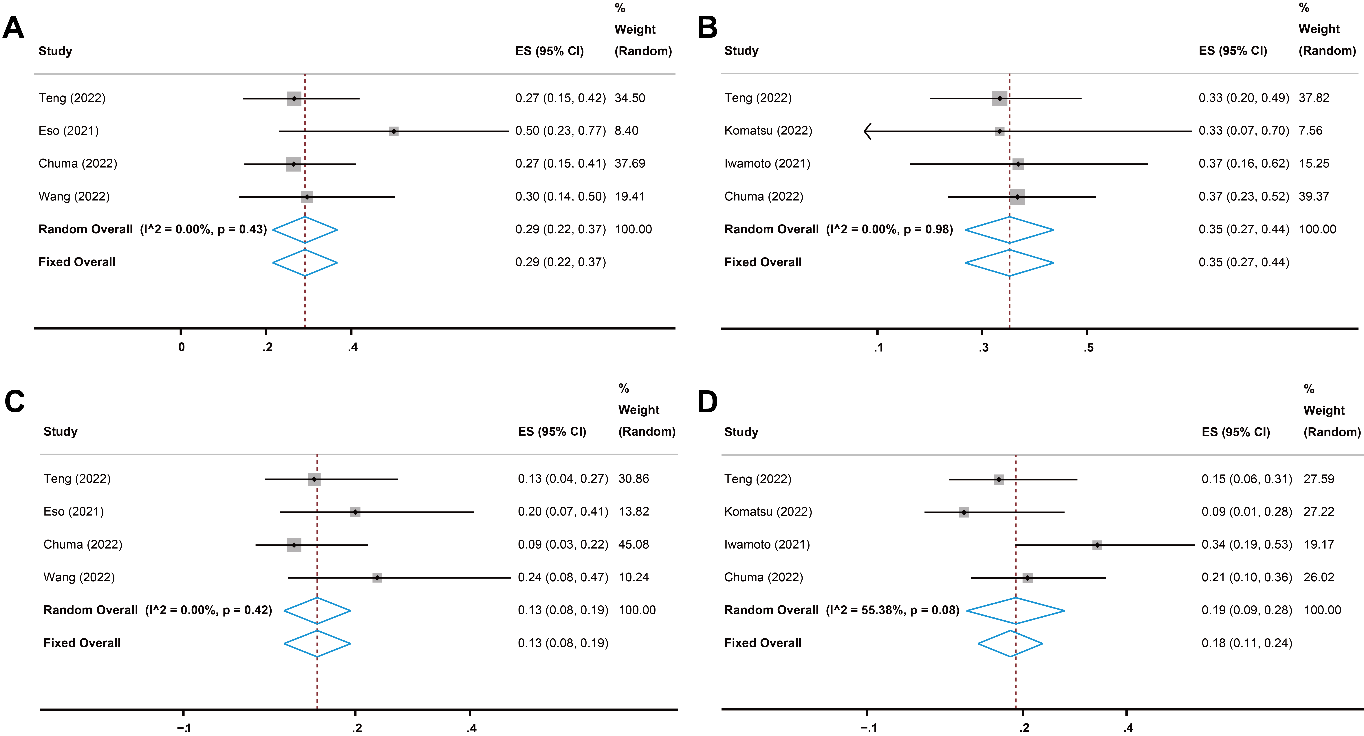


**Supplementary Fig. 1** Pooled OR rates of first-line and second- or later-line treatment with atezolizumab plus bevacizumab. (**A**) Pooled OR rate of first-line treatment based on RECIST. (**B**) Pooled OR rate of first-line treatment based on mRECIST. (**C**) Pooled OR rate of second- or later-line treatment based on RECIST. (**D**) Pooled OR rate of second- or later-line based on mRECIST. OR, overall response; RECIST, Response Evaluation Criteria in Solid Tumors; mRECIST, modified RECIST.

**
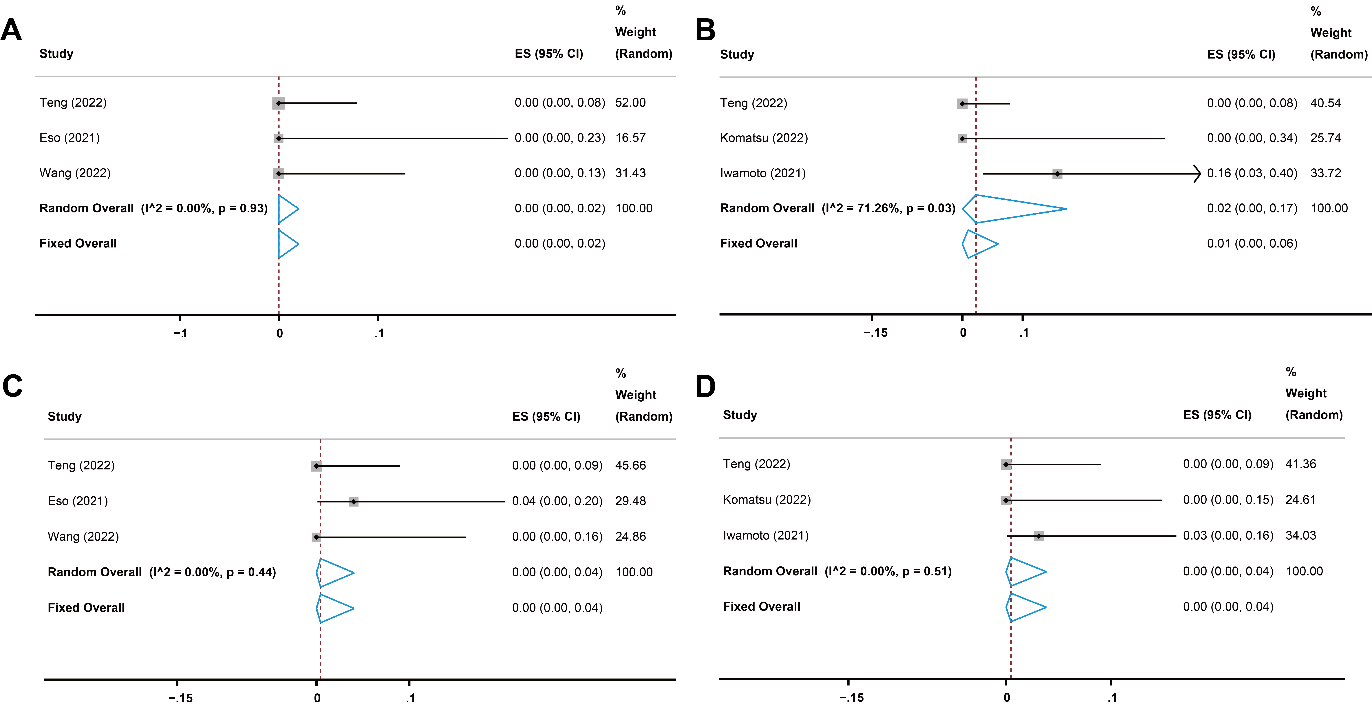
**

**Supplementary Fig. 2** Pooled CR rates of first-line and second- or later-line treatment with atezolizumab plus bevacizumab. (**A**) Pooled CR rate of first-line treatment based on RECIST. (**B**) Pooled CR rate of first-line treatment based on mRECIST. (**C**) Pooled CR rate of second- or later-line treatment based on RECIST. (**D**) Pooled CR rate of second- or later-line based on mRECIST. CR, complete response; RECIST, Response Evaluation Criteria in Solid Tumors; mRECIST, modified RECIST.


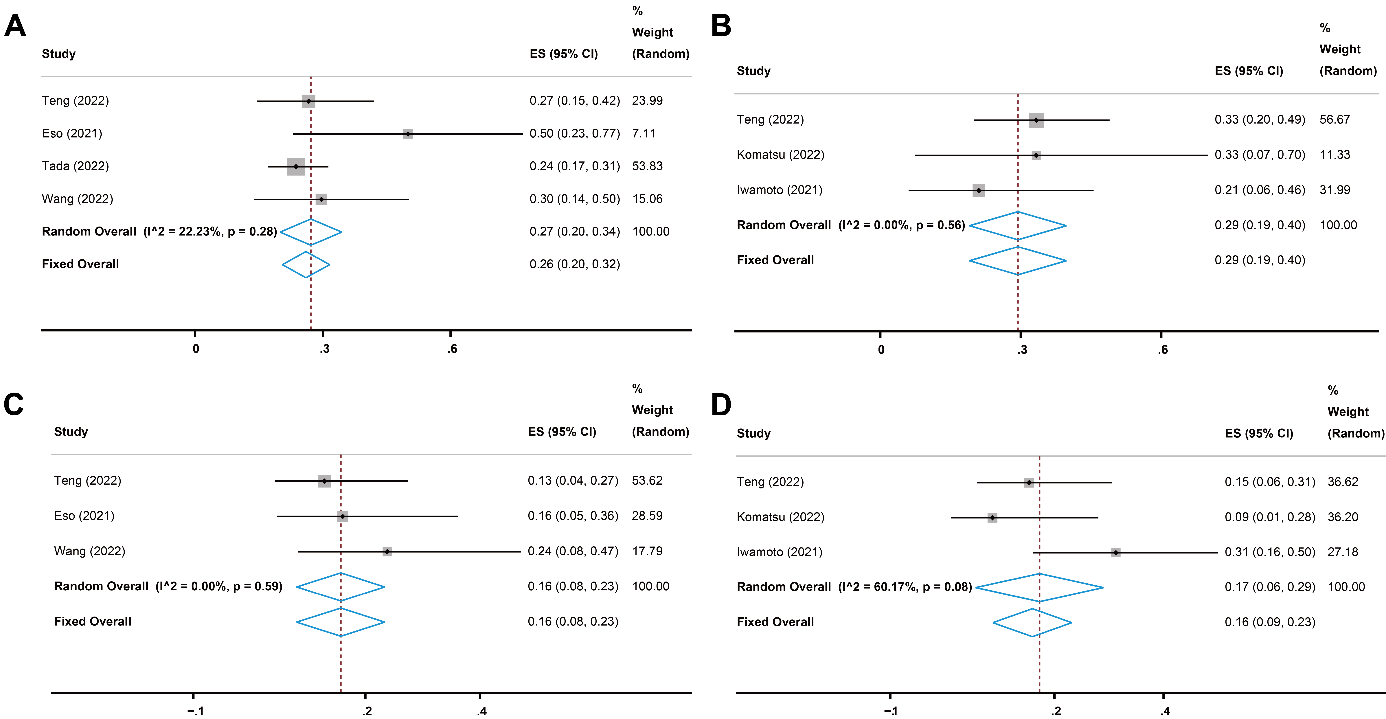


**Supplementary Fig. 3** Pooled PR rates of first-line and second- or later-line treatment with atezolizumab plus bevacizumab. (**A**) Pooled PR rate of first-line treatment based on RECIST. (**B**) Pooled PR rate of first-line treatment based on mRECIST. (**C**) Pooled PR rate of second- or later-line treatment based on RECIST. (**D**) Pooled PR rate of second- or later-line based on mRECIST. PR, partial response; RECIST, Response Evaluation Criteria in Solid Tumors; mRECIST, modified RECIST.


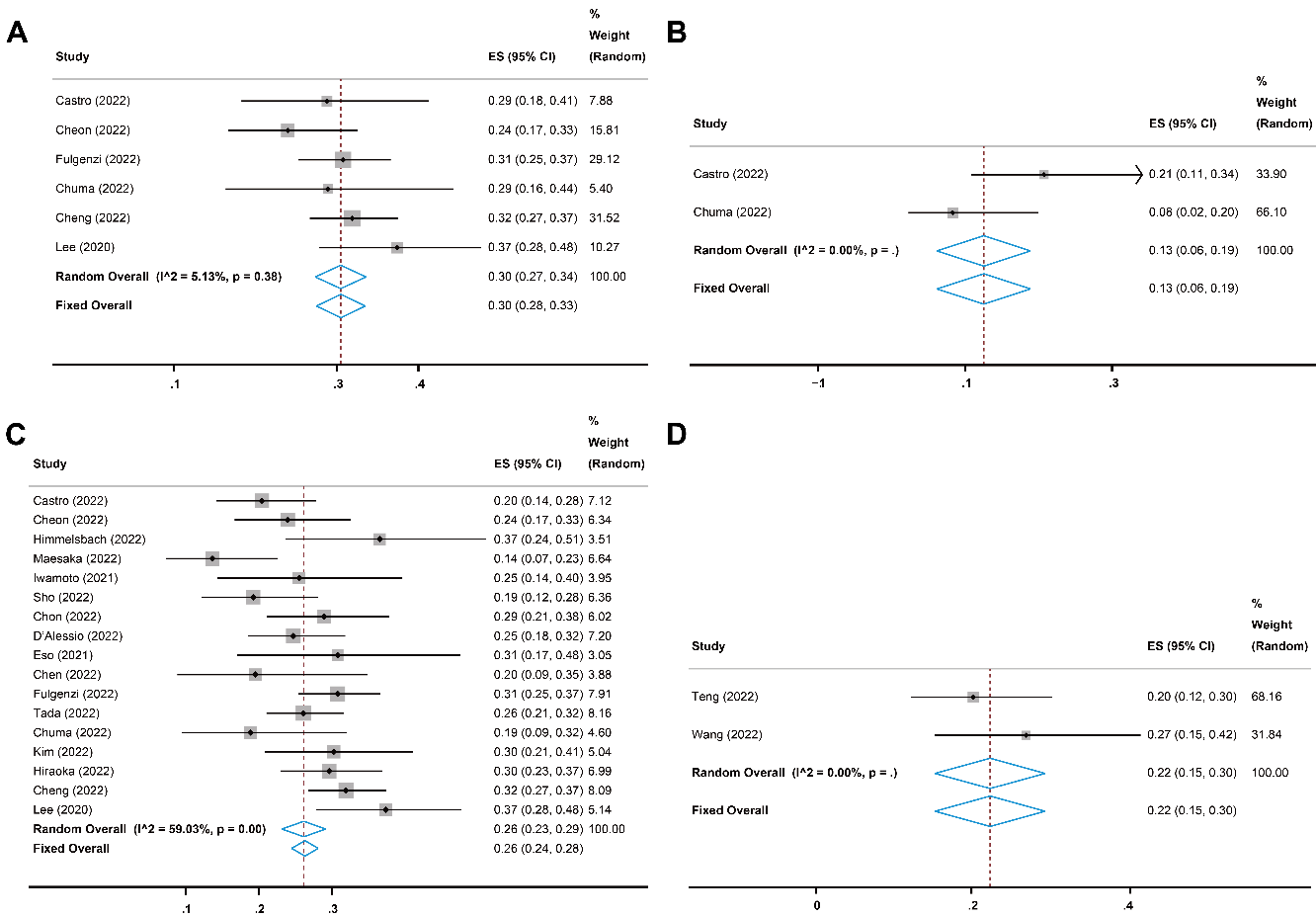
 **Supplementary Fig. 4** Pooled OR rates based on different inclusion criteria and different doses of atezolizumab plus bevacizumab. (**A**) Pooled OR rate of the IMbrave-IN group based on RECIST. (**B**) Pooled OR rate of IMbrave-OUT group based on RECIST. (**C**) Pooled OR rate of standard dose (1200 mg of atezolizumab plus 15 mg/kg of bevacizumab) therapy based on RECIST. (**D**) Pooled OR rate of low dose (1200 mg of atezolizumab plus 5–7.5 mg/kg of bevacizumab) therapy based on RECIST. OR, overall response; RECIST, Response Evaluation Criteria in Solid Tumors.


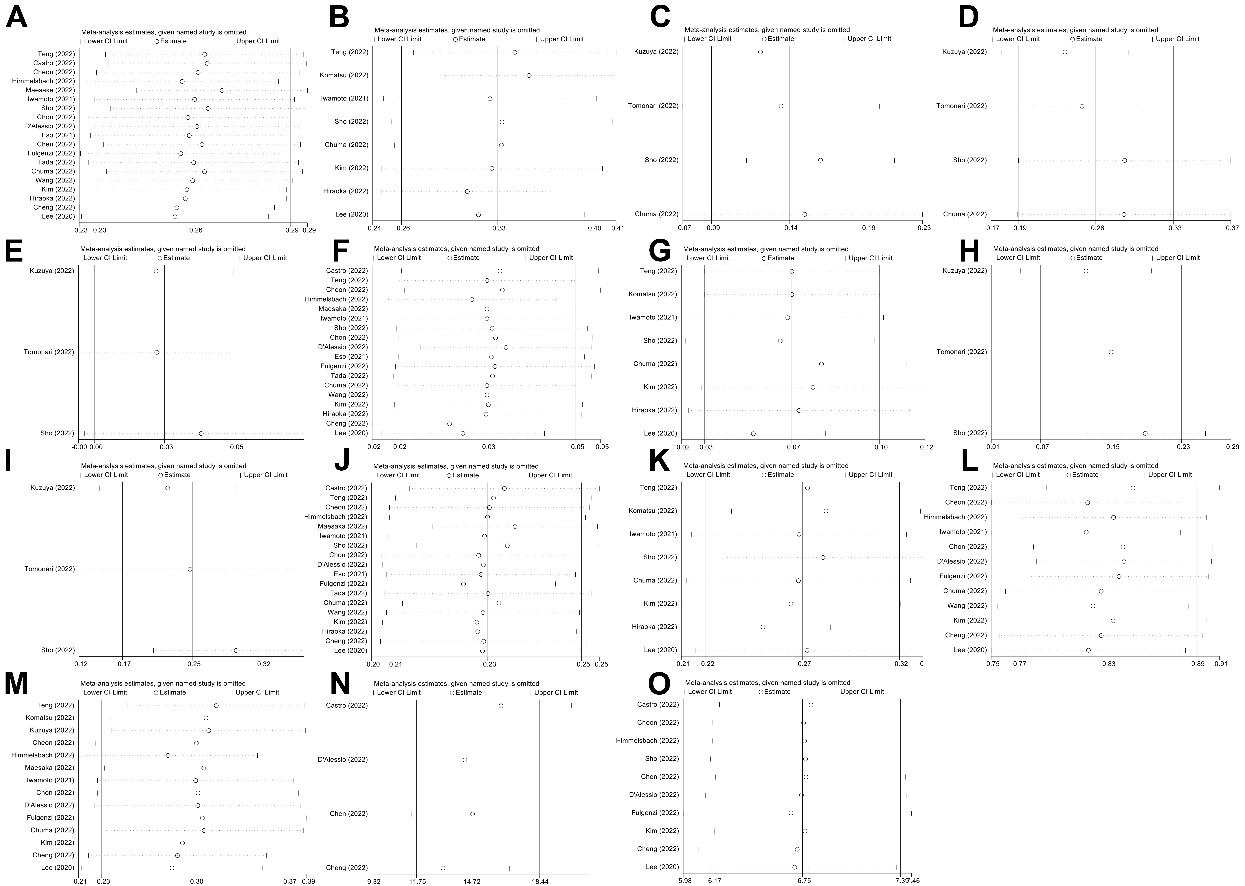


**Supplementary Fig. 5** The results of sensitivity analysis. **(A**) OR rate of non-early treatment based on RECIST. (**B**) OR rate of non-early treatment based on mRECIST. (**C**) OR rate of early treatment based on RECIST. (**D**) OR rate of early treatment based on mRECIST. (**E**) CR rate of early treatment based on mRECIST. (**F**) CR rate of non-early treatment based on RECIST. (**G**) CR rate of non-early treatment based on mRECIST. (**H**) PR rate of early treatment based on RECIST. (**I**) PR rate of early treatment based on mRECIST. (**J**) PR rate of non-early treatment based on RECIST. (**K**) PR rate of non-early treatment based on mRECIST. (**L**) Incidence of all-grade AEs. (**M**) Incidence of grade 3 and above AEs. (**N**) median overall survival. (**O**) median progression-free survival. OR, overall response; CR, complete response; PR, partial response; RECIST, Response Evaluation Criteria in Solid Tumors; mRECIST, modified RECIST. AEs, adverse events.
